# The crosstalk between probiotics and T cell immunity

**DOI:** 10.3389/fimmu.2025.1695840

**Published:** 2025-10-23

**Authors:** Yuanyuan Liu, Xu Cao, Hongwei Liu, Wencan Zhang

**Affiliations:** ^1^ Institute of Biology, Hebei Academy of Sciences, Shijiazhuang, Hebei, China; ^2^ Shanghai Frontiers Science Center for Drug Target Identification and Delivery, and the Engineering Research Center of Cell and Therapeutic Antibody of the Ministry of Education, School of Pharmaceutical Sciences, Shanghai Jiao Tong University, Shanghai, China; ^3^ Shanghai Key Laboratory of Veterinary Biotechnology, School of Agriculture and Biology, Shanghai Jiao Tong University, Shanghai, China

**Keywords:** probiotics, T cell immunity, inflammation, cancer immunotherapy, metabolites

## Abstract

Probiotic supplementation is one of the most widely well-recognized approaches for health maintaining. Distinct probiotics have been experimentally and mechanically investigated for their possible effectiveness in treating autoimmune disease, metabolic diseases, and cancer immune therapy, showing extensive and promising therapeutic potential. Focusing on the T cell mediated responses and diseases, this review specifically aims to elucidate the intricate crosstalk between probiotic microorganisms and T cells, exploring how probiotics modulate the differentiation, activation, proliferation, and functional states of different T cell subsets, such as CD4^+^ T cells and CD8^+^ T cells, thereby influencing the overall immune response and immune homeostasis. By revealing this crosstalk, we aim to uncover the potential of probiotics in immune regulation and disease prevention, providing valuable insights for developing novel therapeutic strategies and preventive measures leveraging the probiotics-T cell axis.

## Highlights

- Probiotic supplementation is a widely adopted and well-established strategy for health maintenance.
- Probiotics modulate CD4^+^ T cell-mediated inflammation and immune homeostasis.
- Probiotics enhance CD8^+^ T cell function in antitumor immunity.
- Engineered probiotics represent novel therapeutic strategies for targeting solid tumors.

## 1 Introduction

The gut microbiota is of paramount importance in shaping the host’s physiology, pathophysiology, and immunology (1–3). Numerous experimental animal studies and a wealth of clinical data have established the pivotal role of the gut microbiome in maintaining digestive health, body weight, inflammation and tumorigenesis. The study of gut microbiota and its interactions with the host have opened up new avenues for research and holds promise for the development of personalized medicine approaches tailored to an individual’s unique microbial profile.

The gastrointestinal tract is a unique environment where the densest population of immune cells resides. These immune cells engage in a dynamic interplay with the microbial community that inhabits the gut, and their communications are bidirectional and involves an intricate network of signaling pathways and molecular mechanisms (4, 5). When certain beneficial microbes are present, they can promote the development and function of immune cells, enhance gut barrier function, and protect against pathogenic infections (6, 7). Conversely, an imbalance in the gut microbiota can lead to chronic inflammation and contribute to the development of various diseases, including autoimmune disorders and certain types of cancer (8, 9). Therefore, understanding the complex relationship between the gut microbiota and the host’s immune system is crucial for maintaining gut homeostasis, improving overall health, as well as developing novel therapeutic strategies.

**Figure 1:**
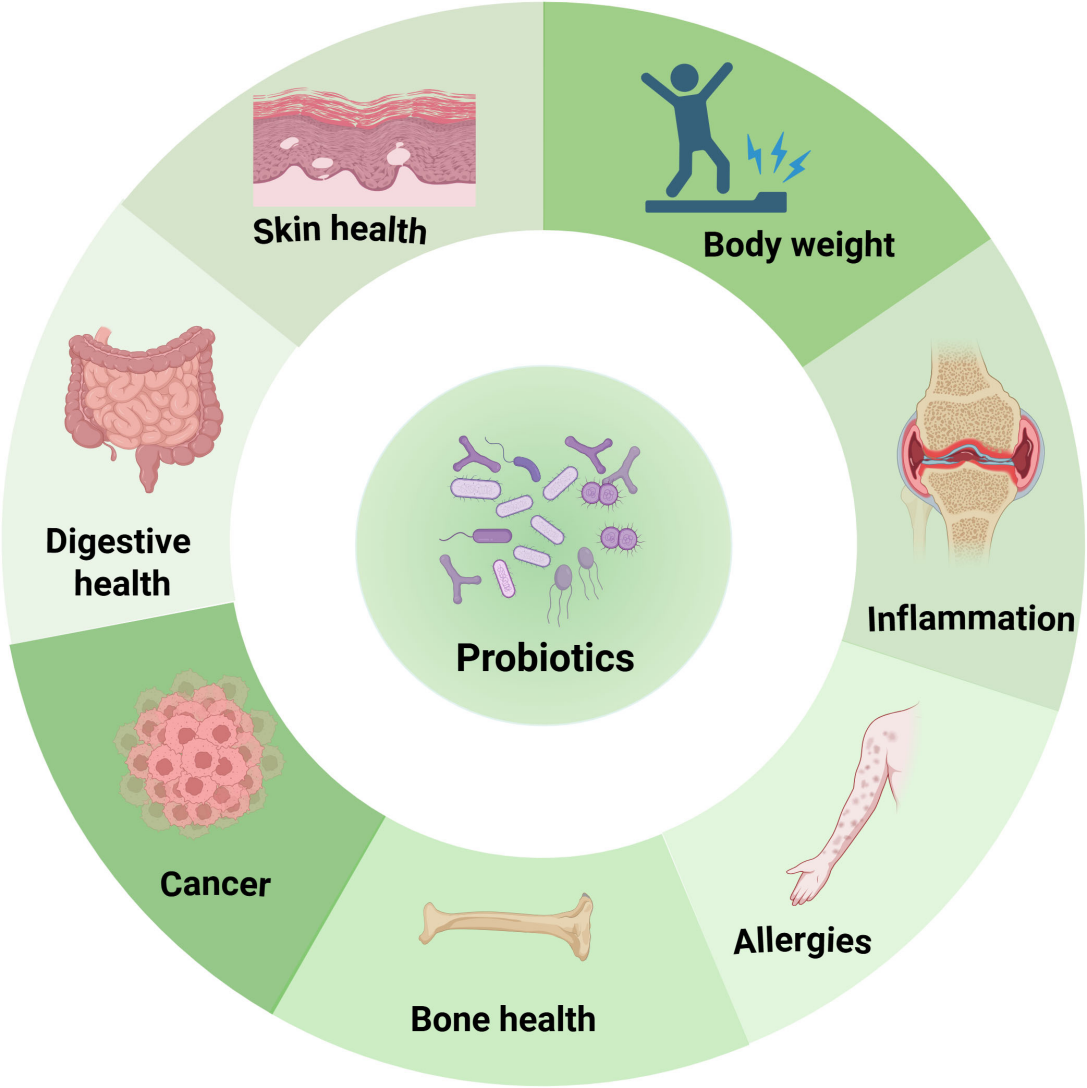
Functionality of probiotics in health maintenance. Probiotics positively influence various aspects of well-being, including digestion, skin health, bone health, cancer prevention, allergy management, inflammation reduction, and body weight regulation.

Currently, probiotic supplementation ranks as one of the most widely adopted and well-recognized approaches for health maintaining by modulating gut microbiota. Probiotics are live microorganisms, usually bacteria or yeasts, that confer health benefits on the host when consumed in adequate amounts (10). They primarily reside in the gut and can positively influence the host’s health. Also, they can be found in fermented foods like yogurt, kefir, sauerkraut, kimchi, miso, tempeh, and kombucha, among others (11–13). The most common strains currently available as probiotics and possessing beneficial health effects are *Lactobacillus, Bifidobacterium, Bacillus, Saccharomyces boulardii*, *Streptococcus thermophilus* and *Pediococcus pentosaceu* (14). By introducing into the gastrointestinal tract, probiotics can help counteract the overgrowth of harmful pathogens, reduce inflammation, and improve overall health (15, 16). It has been well established that the probiotics play pivotal roles in maintaining digestive health and body weight, bone quality, alleviating inflammation, immune disorders and tumorigenesis, as shown in **Figure 1**, underscoring its therapeutic potential.

Emerging research has highlighted the profound influence of probiotics on the immune system, particularly in the regulation of T cell immunity, by themselves or by their byproducts (17–20). T cells constitute central players within the adaptive immune system, orchestrating cell-mediated immune responses essential for maintaining host health and defending against a spectrum of threats (21, 22). By influencing the differentiation, activation, proliferation, and functional states of different T cell subsets, including CD4^+^ T cells and CD8^+^ T cells, probiotics show great potential in immune regulation and disease prevention.

This review seeks to elucidate the intricate crosstalk between probiotic microorganisms and T cell immunity, while providing valuable insights for the development of novel therapeutic strategies and preventive interventions targeting the probiotics-T cell axis. By synthesizing mechanistic findings from *in vitro*, animal, and human studies, we delineate the molecular pathways through which probiotics orchestrate T cell responses and restore immune homeostasis. We further delve into the translational potential of probiotics for managing autoimmune diseases, metabolic syndrome, and cancer immunotherapy, emphasizing the emerging “probiotics-T cell axis” as a promising therapeutic target for future research and clinical applications.

## 2 Association of the gut microbiome with T cell immunity

**Figure 2:**
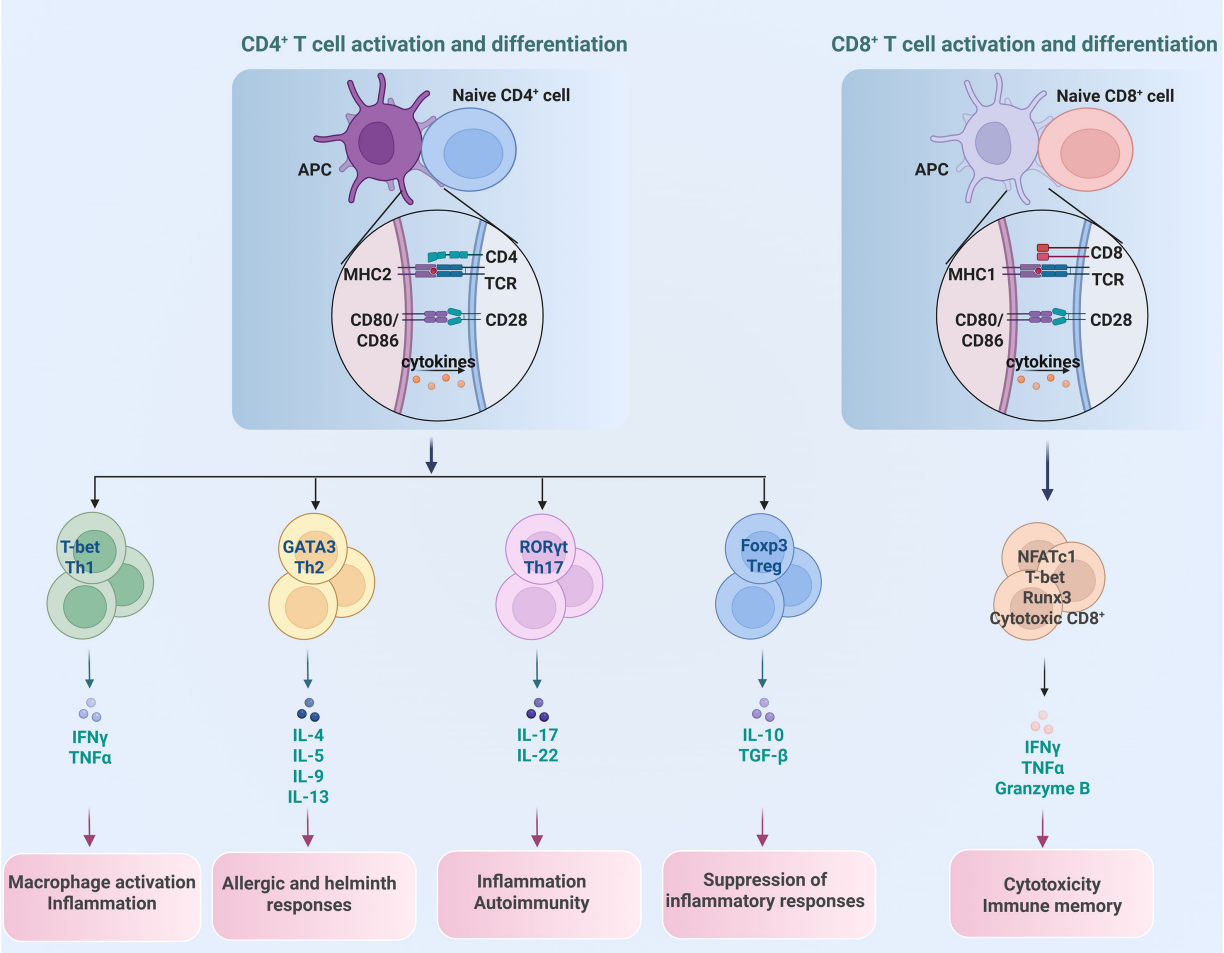
Differentiation of different T cell subset and their distinct function. CD4^+^ T cells differentiate into subsets of Th1, Th2, Th17 and Treg, each with unique roles in promoting or regulating immune reactions, while CD8^+^ T cells primarily function as cytotoxic T cells to eliminate cancer cells.

T cells are the principal cell components of the adaptive immune system. They are developed from bone marrow-derived thymocyte progenitors in the thymus, and broadly grouped into CD4^+^ and CD8^+^ αβ T cells and a rear populations of γδ T cells as well as natural killer T (NKT) cells (23). αβ T cells recognize antigens that are presented by major histocompatibility complex (MHC) molecules on antigen-presenting cells (APCs). When the T cell receptor (TCR) engages its cognate peptide-MHC complex, costimulatory signals and cytokines, naïve CD4^+^ and CD8^+^ T cells undergo activation, clonal expansion, and lineage-specific differentiation. The resulting effector populations execute their distinct functions of eliminating infected cells, secreting immunoregulatory cytokines, and regulating the broader immune responses (24), as shown in **Figure 2**.

Upon recognition of peptide-MHC class II complexes on APCs, naïve CD4^+^ T cells embark on differentiation programs that yield functionally distinct subsets, such as T helper 1 (Th1), Th2, Th17 and T regulatory (Treg) cell, with unique surface phenotypes, signature cytokines, and lineage-specifying transcription factors (TFs) (25, 26). Th1 cells are the major participants in protecting hosts against intracellular bacteria and viruses by producing the pro-inflammatory cytokine IFNγ. Th2 cells, producing cytokines of IL-4, IL-5, and IL-13, protect the host against helminth infections, facilitate tissue repair, as well as contribute to asthma and allergy. Th17 cells, characterized by expression of cytokines IL-17, IL-21, IL-22, and IL-23, and steroid receptor–type nuclear receptor RORγt as the master TF, contribute to protection against extracellular pathogens as well as chronic inflammation and autoimmune diseases (27, 28).Treg cells, on the other hand, are a specialized CD4^+^ T cell subset that suppress excessive immune responses, preventing autoimmune reactions and maintaining immune tolerance to self-antigens (29). Treg cells are characterized by the master TF Foxp3, as well as high expression of IL-2 receptor alpha chain (IL-2Rα, CD25), inhibitory cytokines IL-10, TGF-β, and IL-35 (30, 31). Based on their developmental origin, there are two subsets of Treg cells: thymic Treg (tTreg) cells that derive from thymus, and induced Treg (iTreg) cells that differentiate from conventional CD4^+^ T cells in the periphery after antigen stimulation and in the presence of TGF-β (32).

CD8^+^ T cells are another major T cell subset that play critical roles in fighting against intracellular pathogens as well as eliminating malignant cells (33). Upon recognition of peptide-MHC class I complexes on APCs, naïve CD8^+^ T cells undergo robust expansion to give rise to effector and memory T cells. Effector CD8^+^ T cells can directly induce target cell death by secreting IFNγ, TNFα and granzymes B. Memory CD8^+^ T cells provide rapid and strong protection upon antigen reencounter, which is critical for effective and long-term protective immunity (34, 35).

The precise regulation of distinct T cell subsets, including activation, proliferation and functional modulation, is essential for maintaining immune homeostasis. Any disruption in these balances, such as overactivation of pro-inflammatory T cell subsets (e.g., Th1 or Th17) without sufficient regulatory control, can lead to immune-mediated pathologies like autoimmune diseases, chronic inflammation, or excessive tissue damage (36). Conversely, suppression of effector T cell responses without adequate inflammation can result in persistent infections or tumor formation (37).

**Figure 3:**
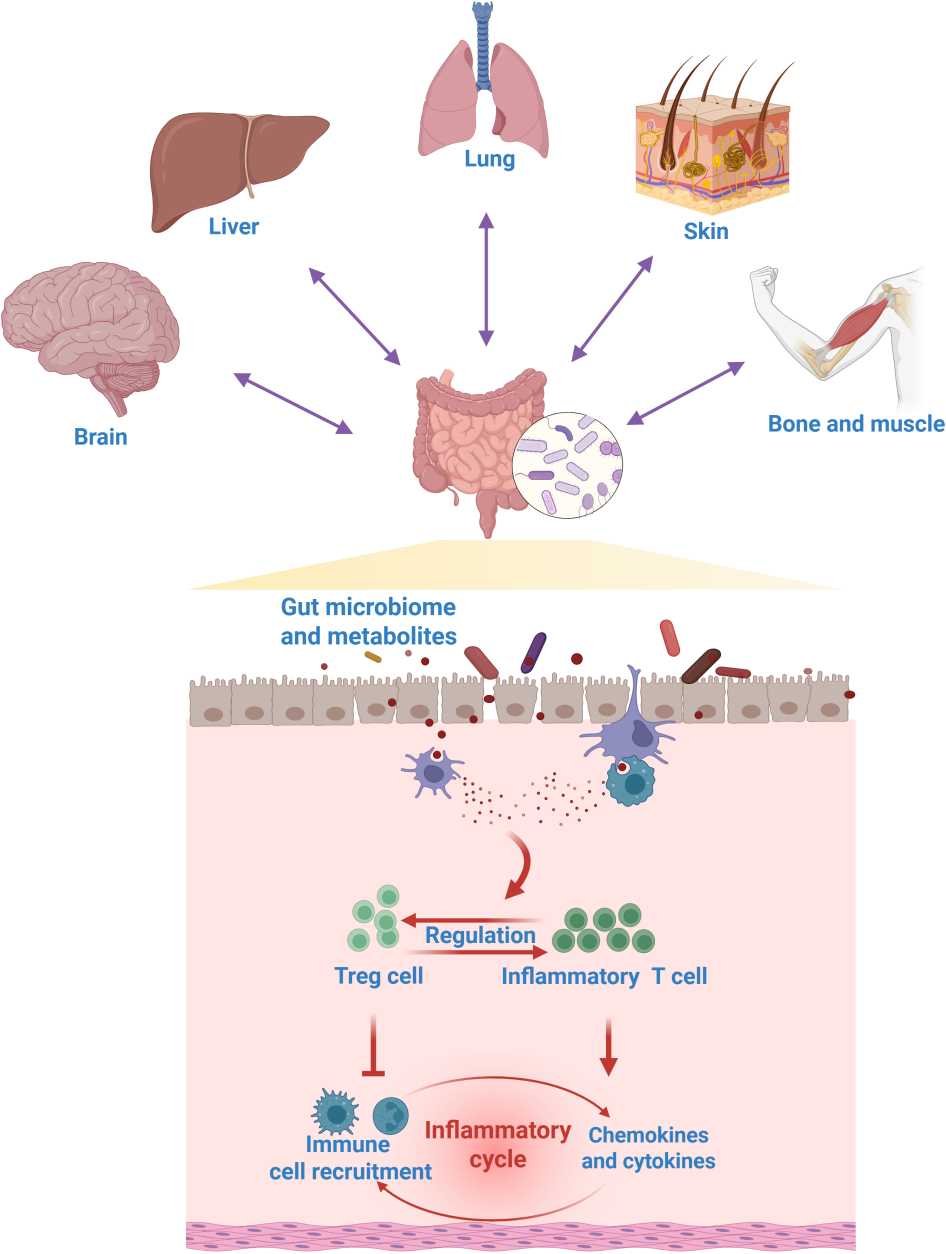
The role of gut microbiome in regulating T cell immunity. The gut microbiome acts as a key modulator of systemic T cell immunity, modulating a wide spectrum of gut-systemic immune axis.

The gut microbiome plays a fundamental role in shaping and modulating host T cell immunity, establishing a critical dialogue at the mucosal interface. Through various mechanisms, commensal bacteria directly influence the differentiation, expansion, and function of diverse T cell subsets. For instance, certain microbial species, such as segmented filamentous bacteria, are potent inducers of Th17 cells in the intestine, which are crucial for defending against extracellular pathogens (38). Conversely, other bacteria, like specific *Clostridia* strains, promote the generation of Treg cells, maintaining immune tolerance and preventing aberrant inflammation (39). Moreover, metabolites derived from the gut microbiota serve as critical immune modulators. Specifically, short-chain fatty acids (SCFAs), key byproducts of gut bacterial fermentation, modulate immunometabolic pathways to suppress Th17 cell development, preventing over-inflammation (40). Tryptophan breakdown products, such as indole derivatives, enhance the abundance of intraepithelial CD4^+^CD8^+^ T cells. Additionally, bacterial polysaccharides actively promote the differentiation of Treg cells for restraining excessive immune responses (8). A balanced gut microbiome fosters a robust and appropriately regulated T cell repertoire, not only within the gut but also systemically, thereby influencing the host’s susceptibility to a wide range of conditions, from inflammatory bowel disease and autoimmunity to responses to cancer immunotherapy and infectious diseases. Thus, the interaction between gut microbiome and T cell immunity represents a crucial dimension of their functionality, underscoring their significance as immune regulators and health guardians, as shown in **Figure 3**.

Supplementation with probiotics and prebiotics can induce alterations in the gut microbiome and confer health benefits on the host. The effects of probiotics vary depending on type and dose as well as on their interaction with the host in different ways. Some exhibit direct antibacterial action via the production of substances such as bacteriocins, hydroperoxides, lactic acid, and defensins (41–43). Others exhibit non-immunological action such as competing with pathogens for nutrients, increasing mucus production, changing intestinal pH, by promoting the formation of tight junctions, or enhancing tissue repair processes, thereby reducing intestinal mucosal permeability (7, 44, 45). Moreover, probiotics and their metabolites can influence the host’s immune response, modulating both innate and adaptive immunity (15, 46). The detailed information is summarized in **Table 1** and will be discussed in the subsequent sections.

**Table 1:** Studies of different probiotic strains effects in modulating T cell immunity.

T cell functionality	Representative examples	Molecular mechanisms	Immunomodulatory effect	Models	References
T cell development and maturation	*Mixture of L. acidophilus* and *B. bifidum*	N.A.	Promoted the maturation of T cells in thymus	Mouse models.	(47)
	*L. reuteri*	Altered DNA methylation patterns in CD4^+^ T cells.	Promoted the maturation of CD4^+^ T cells in thymus	Mouse models.	(48)
	Mixture of *L. rhamnosus* and *B. lactis*	Increased in type 1 conventional dendritic cells leaded to a boost in IAV- specific, IFNγ-producing effector CD8^+^ T cells in neonates.	Enhanced influenza A/PR8 virus (IAV) clearance in neonates	Mouse models.	(49)
CD4^+^ T cell mediated inflammation	Mixture of *B. longum, L. acidophilus* and *E. faecalis*	The increased production of spermidine potentiates Th1 cell immunity via autophagy activation.	Promoted HBV clearance through autophagy-driven enhancement of IFNγ^+^CD4^+^ T cell immunity.	Mouse models.	(50)
	*B. bifidum*	Enhanced Th1 responses and IFNγ producing.	Protected mice from the challenge with H1N1 influenza virus	Mouse models.	(51)
	*L. rhamnosus* M21	Promoting Th1 cytokines of IL-2 and IFNγ.	Increase the survival of mice after influenza virus challenge	Mouse models.	(52)
	*L. intestinalis*	Promoted retinoic acid synthesis further triggered epithelial gene SAA1, SAA2, and C/EBPA alteration and downregulated RORγt^+^ Th17 cells.	Attenuated colitis.	Mouse models.	(53)
CD4^+^ T cell mediated immune tolerance	*B. adolescentis*	Modulating the Treg/Th2 response by decreasing proinflammatory cytokines such as TNFα, IL-6, IL-1β, IL-18, IL-22, IL-9 and increasing anti-inflammatory cytokines IL-10 and IL-4 in mice colitis model.	Improved chronic colitis.	Mouse models.	(54)
	*L.rhamnosus* GG	N.A.	Improved survival and reduced aGVHD.	Mouse models.	(55)
	Mixture of *L.rhamnosus*, *L.casei*, *L.bulgaricus*, *L.acidophilus*, *B. breve*, *B. longum*, and *S. thermophilus*	Increasing the induction of Treg cells.	Lower the frequency and intensity of aGVHD.	Patients.	(56)
	*L. rhamnosus* GG	Enhanced the Th17/Treg balance by effectively downregulating the expression of RORγt and upregulating the expression of Foxp3.	Improved osteoporosis in ovariectomized (OVX) rats	Rat models.	(18)
	Mixture of *B. bifidium CBT BF3, B. breve* CBT BR3*, L. acidophilus* CBT LA1*, L. plantarum* CBT LP3*, L. rhamnosus* CBT LR5*, L. lactis* CBT SL6, and *S.thermophilus* CBT ST3	Induced generation of CD4^+^Foxp3^+^ T cells and decreased the levels of inflammatory cytokines related to Th1 and Th2 cells.	Alleviated AD symptoms	Mouse models.	(57)
	*W. cibaria* WIKIM28	Increased Treg cells and inhibited proinflammatory cytokines.	Alleviate AD symptoms.	Mouse models.	(58)
	*L. plantarum* CJLP133	Increased Treg cells and inhibited proinflammatory cytokines.	Alleviate AD symptoms.	Patients.	(59)
CD8^+^ T cell mediated antitumoral immunity	*L. plantarum* L168	Produced more indole-3-lactic acid, which enhanced H3K27ac binding at the enhancer regions of IL-12a, thereby boosting IL-12a production in dendritic cells and priming CD8^+^ T cell immunity against tumor growth. Indole-3-lactic acid transcriptionally inhibited Saa3 expression, altering cholesterol metabolism of CD8^+^ T cells.	Mitigated intestinal inflammation, tumor growth, and gut dysbiosis.	Mouse models.	(60)
	*L. bulgaricus* OLL1073R-1	phosphorylated structure in EPS-R1 engages a lysophosphatidic acid receptor on CD8^+^ T cells, which further induced the expression of CCR6 in CD8^+^ T cells. increased infiltration of CCR6^+^ CD8^+^ T cells	Augmented antitumor effects of anti-CTLA-4 or anti-PD-1 monoclonal antibody against CCL20-expressing tumors.	Mouse models.	(20)
	*B. coccoides*	Elevated the level of trigonelline, which further downregulated β-catenin expression and promoted the infiltration and antitumor activity of CD8^+^ T cells.	Suppressed tumor growth and enhanced infiltration of CD8^+^ T cells into the tumor microenvironment	Mouse models	(61)
	*L.reuteri*	Promoted the release of the dietary tryptophan metabolite I3A, which locally stimulated the production of IFNγ by CD8^+^ T cells.	Enhanced the efficacy of immune checkpoint inhibitors.	Mice model and patients	(17)
	*R. intestinalis*	Through the production of butyrate to activate cytotoxic CD8^+^ T cells, enhanced the production of granzyme B, IFNγ and TNFα. Butyrate directly binds to TLR5 on CD8^+^ T cells, activating NF-κB signaling to enhance T cell activity	Suppressed tumor growth in orthotopic MC38 and CT26 models.	Mouse models	(19)
	*B. pseudolongum*	Supplying L-arginine and promoted CD8^+^ T-cell differentiation into memory cells.	Potentiated anti-CTLA-4 therapy in orthotopic CRC models.	Mouse models.	(62)
	*L. gallinarum*	By reducing Foxp3^+^ Treg intratumoral infiltration, and enhancing effector function of CD8^+^ T cells. *L. gallinarum*-derived indole-3-carboxylic acid (ICA) was identified as the functional metabolite. Mechanistically, ICA inhibited indoleamine 2,3-dioxygenase (IDO1) expression, therefore suppressing kynurenine (Kyn) production in tumors. ICA also competed with Kyn for binding site on aryl hydrocarbon receptor (AHR) and antagonized Kyn binding on CD4^+^ T cells, thereby inhibiting Treg differentiation *in vitro* and *in vivo*.	Improve anti-PD1 efficacy in mouse MC38 and CT26 CRC tumorigenesis models	Mouse models.	(63)
	*L. acidophilus*	Increased CD8^+^ T cells and effector memory T cells (CD44^+^CD8^+^CD62L^+^) and decreased immunosuppressive Tregs and M2 macrophages (F4/80^+^CD206^+^).	Enhance the antitumor activity of CTLA-4 blockade when combined with an anti-CTLA-4 antibody in syngeneic BALB/c mouse models of CRC.	Mouse models.	(64)
	*L. rhamnosus* Probio-M9	Accumulated butyric acids in the gut, and blood-derived α-ketoglutaric acid, N-acetyl-l-glutamic acid and pyridoxine. These molecules together dampened the function of Treg cells while enhancing the infiltration and activation of CD8^+^ T cell.	Boosted the anti-PD-1-based tumor inhibition by enhancing cytotoxicity of CD8^+^ T cells and suppressing Treg cells.	Mouse models.	(65)
	*C. cateniformis*	Decreased PD-L2 expression on dendritic cell and its interaction with repulsive guidance molecule b (RGMb).	Improved the efficacy of PD-1 inhibitors.	Mouse models.	(66)
Novel therapeutic approaches for T cell mediated solid tumor targeting	Engineered *E. coli* Nissle 1917	Efficiently channel ammonia toward arginine synthesis through deleting the arginine repressor gene (ArgR) and integrating the N-acetylglutamate synthase gene (ArgA).	Increased intratumoral L-arginine, expanded the population of tumor-infiltrating T cells, and synergized with PD-L1 blocking antibodies tumors clearance.	Mouse models.	(67)
	Engineered *E. coli-*based system	Local delivery of PD-L1 and CTLA-4 antibodies.	Promoted tumor regression and increased activated T cells and T cell memory populations.	Mouse models.	(68)
	Engineered *E. coli*	Delivers synthetic antigens to the tumor microenvironment, effectively "tagging" the tumor cells for CAR-T cell detection.	Promoted tumor cell killing.	Mouse models and patients.	(69)

## 3 Probiotic supplementation modulates T cell development and maturation

T cell development and maturation during early life are of paramount importance. The proper development and maturation of T cells are crucial for the immune system to effectively combat pathogens and maintain self-tolerance, ensuring the body’s defense function without harming healthy tissues. Previous research found that maternal gut microbiota composition contributes to the status of the neonatal T cell immune system and immune response during early life (70–72).

Furthermore, probiotic supplementation during pregnancy also has effects both on T cell development and maturation. A recent study found that perinatal probiotic exposure profoundly influenced immune cell composition in the intestine, liver and lungs of newborn mice (47). In detail, when pregnant mice were orally treated with a combination of *Lactobacillus acidophilus* (*L. acidophilus*) and *Bifidobacterium bifidum* (*B. bifidum*) from mid-pregnancy until the offspring were harvested, a reduction of myeloid and B cells and induction of T cells were found in the probiotic treated animals’ organs at weaning. Moreover, in the same experimental settings, probiotic exposure had an effect on T cell development in the thymus by increasing the proportion of CD4^+^ and CD8^+^ single positive populations at the weaning time. Thus, prenatal exposures to *L. acidophilus* and *B. bifidum* promoted the maturation of T cells in thymus. This finding was supported by another independent study, in which maternal *L. reuteri* supplementation during pregnancy was found to affect T cell maturation of neonates at birth, by altering DNA methylation patterns in CD4^+^ T cells towards enhanced immune activation (48).

The influence of perinatal probiotic exposure extends beyond T cell maturation to also impact T cell function in early life. Another recent study demonstrated that perinatal exposure to *Lacticaseibacillus rhamnosus* (*L. rhamnosus*) or *B. animalis subsp. Lactis* (*B. lactis*) enhances influenza A/PR8 virus (IAV) clearance in neonates (49). The mechanism involves an increase in type 1 conventional dendritic cells (cDC1) in the lymph nodes following supplementation. This leads to a boost in IAV- specific, IFNγ-producing effector CD8^+^ T cells in neonates and IAV-specific resident memory CD8^+^ T cells in adulthood, aligning with greater protection for offspring during secondary infections. Notably, while no gut microbiota disruption was observed in offspring or mothers, untargeted metabolomic analysis revealed that neonatal plasma metabolites were altered. Further research indicated that genistein and 3-(3-hydroxyphenyl) propionic acid can replicate viral clearance or cDC1 activation in neonates exposed to IAV. In summary, maternal supplementation with *L. rhamnosus* or *B. lactis* imparts specific metabolomic modulations to neonates, bolstering CD8^+^ T cell-mediated immune protection against IAV infection.

## 4 Delicate crosstalk between probiotics and CD4^+^ T helper cells

### 4.1 Probiotic supplementation regulates CD4^+^ T cell mediated inflammation

**Figure 4:**
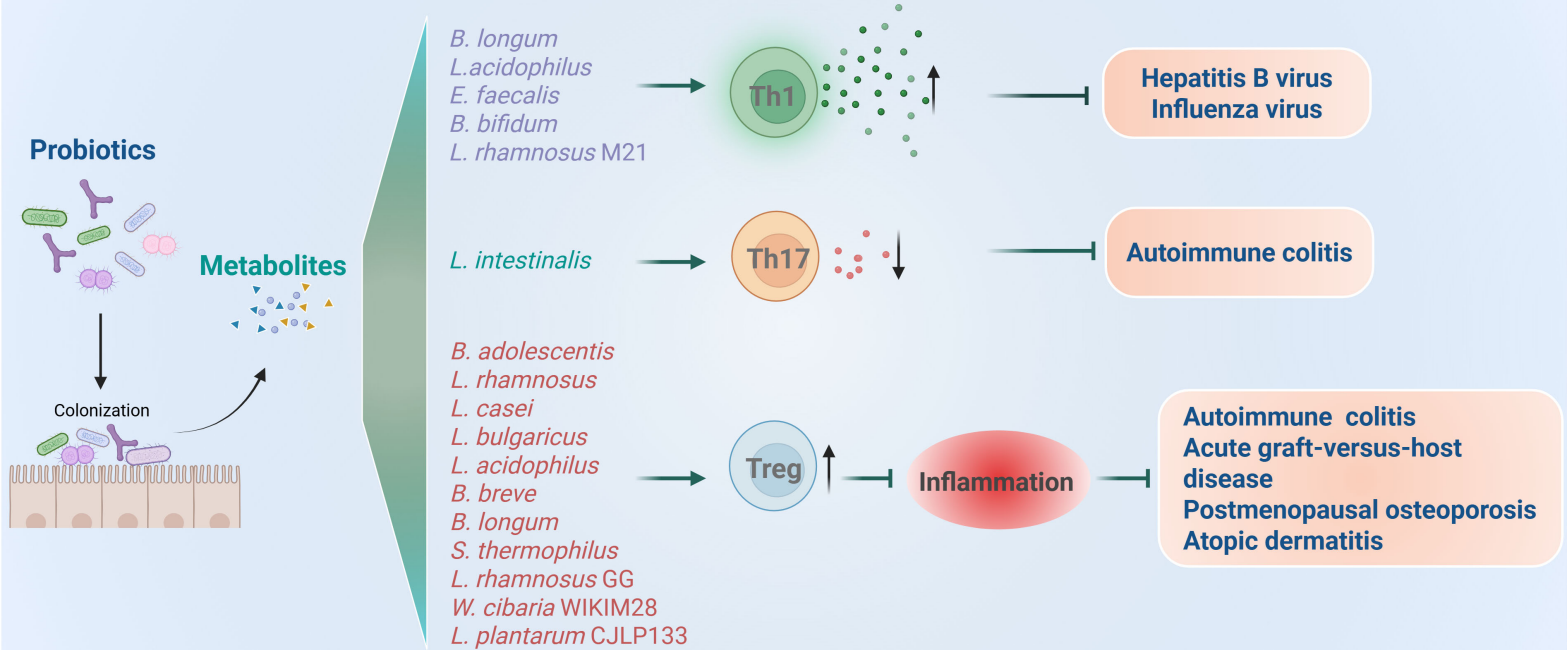
Roles of probiotics in regulation of CD4^+^ T cell mediated immune responses and related diseases. Probiotics regulate the inflammation induced by Th1 and Th17 and immune inhibition induced by Treg.

CD4^+^ T helper cell-mediated inflammation is critical for effective immune responses in protection against infection. However, dysregulated CD4^+^ T cell responses can induce immunopathology, including autoimmune disorders. For instance, over-differentiation toward Th17 cells contributes to the progression of multiple sclerosis, rheumatoid arthritis, and inflammatory bowel disease through producing proinflammatory cytokines including IL-17, IL-22 and IL-23 (73, 74). Notably, a number of probiotics and their metabolites can modulate CD4^+^ T cell differentiation, demonstrating significant roles in regulating inflammation, as shown in **Figure 4**.

It was found that supplementation of probiotic mixture containing *B. longum*, *L. acidophilus*, and *Enterococcus faecalis* (*E. faecalis*) can enhance antiviral immunity by promoting the release of proinflammatory cytokines (50). In the case of chronic hepatitis B virus (HBV) infection, using both specific-pathogen-free (SPF) and germ-free mouse models, researchers observed significant suppression of HBV replication following probiotic administration. They further revealed that spermidine produced by probiotic mixtures potentiates Th1 cell immunity via autophagy activation. More importantly, preliminary clinical data from HBV patients indicate that such probiotic intervention may accelerate viral clearance. Collectively, these findings highlight the therapeutic potential of probiotic mixture of *B. longum*, *L. acidophilus*, and *E. faecalis* and spermidine in promoting HBV clearance through autophagy-driven enhancement of IFNγ^+^CD4^+^ T cell immunity, suggesting a novel strategy for achieving functional cure in HBV patients. Besides HBV, probiotics are also protective from influenza virus by promoting Th1 response. When the mice were treated with *B. bifidum*, their Th1 responses and IFNγ producing were enhanced, which protected mice from the challenge with H1N1 influenza virus (51). Similarly, oral administration of *L. rhamnosus* M21 was also found to increase the survival of mice after influenza virus challenge through promoting Th1 cytokines of IL-2 and IFNγ (52). Collectively, probiotics mentioned above can enhance antiviral immunity by increasing the secretion of proinflammatory cytokines and Th1 responses.

However, over-secreted proinflammatory cytokines will induce autoimmune disorders and autoimmune diseases, including rheumatoid arthritis, lupus, type 1 diabetes, multiple sclerosis, and inflammatory bowel disease (75–77). Recent studies found that probiotic supplementation has potential therapeutic effects for treating autoimmune diseases by suppressing inflammation. For instance, a recent work identified *L. intestinalis* as a novel protective bacterium that promotes intestinal homeostasis, as *L. intestinalis* supplementation attenuated colitis and downregulated the levels of RORγt^+^ Th17 cells in the colon (53). Mechanically, *L. intestinalis* enhanced retinoic acid synthesis through collaborative metabolism with the host, and retinoic acid triggers epithelial gene alteration, including SAA1, SAA2, and C/EBPA, to downregulate RORγt^+^ Th17 cells.

### 4.2 Probiotic supplementation regulates immune tolerance

Different from proinflammatory CD4^+^ T helper cells, Treg cells are immune suppressive that play a critical role in maintaining immune homeostasis and preventing excessive immune responses. Some probiotics have been found to modulate the formation of Treg cells, thereby helping to control overactivated immune responses in chronic inflammatory conditions.

Studies found that *Bifidobacteria adolescentis* (*B. adolescentis*) improves chronic colitis by modulating the Treg/Th2 response and reshaping the gut microbiota (54). Specifically, *B. adolescentis* administration reduced diarrhea scores and spleen weight, increased colon length, and lowered cumulative histological grades by decreasing proinflammatory cytokines such as TNFα, IL-6, IL-1β, IL-18, IL-22, IL-9 and increasing anti-inflammatory cytokines IL-10 and IL-4 in mice colitis model. Mechanically, the colon lamina propria had more Treg and Th2 cells, curbing excessive gut inflammation. Overall, *B. adolescentis* treatment stimulated a protective Treg/Th2 response in chronic colitis, rendering it a promising adjunctive strategy for treating inflammatory bowel disease.

Acute graft-versus-host disease (aGVHD) is another type of disease induced by immune imbalance. It represents a major limitation to successful outcomes and a leading cause of mortality following allogeneic hematopoietic stem cell transplantation (allo-HSCT) (78, 79). This condition develops when donor-derived T cells become activated, producing proinflammatory cytokines such as IFNγ, TNFα and IL-17, which mediate damage to host tissues. Notably, clinical evidence indicates that higher frequencies of Treg cells in allo-HSCT recipients correlate with reduced aGVHD risk, whereas diminished Treg levels exacerbate aGVHD susceptibility (80–82). As early as 2004, researchers found that oral administration of *L. rhamnosus* GG before and after transplantation results in improved survival and reduced aGVHD in mice models. However, they did not figure out the detailed mechanism (55). A recent clinical trial study has revealed that using synbiotics daily, before and during the conditioning regimen for patients undergoing allo-HSCT, could lower the frequency and intensity of aGVHD by increasing the induction of Treg cells post-transplantation (56). Specifically, each synbiotic capsule contained a high level (10^9^ CFU) of seven bacterial strains including *L. rhamnosus*, *L. casei*, *L.bulgaricus*, *L. acidophilus*, *Bifidobacterium breve*, *B. longum*, and *Streptococcus thermophilus* (*S. thermophilus*), that are safe and beneficial to the human body, plus fructooligosaccharides (FOS) as a prebiotic.

Besides, as our comprehension of the gut-bone axis deepens, more studies are exploring treatments for postmenopausal osteoporosis by modulating gut microbes with probiotics supplementation (9, 83). Clinical research has shown that the gut microbiome significantly impacts bone quantity, quality, and strength (84, 85). Moreover, the Th17/Treg balance and related inflammatory factors are closely linked to bone metabolism dysregulation (86). A recent study found that *L. rhamnosus* GG (LGG) improves osteoporosis in ovariectomized (OVX) rats (18). In the OVX rat model, TNFα and IL-17 expression rose in the colon and bone marrow, while TGFβ and IL-10 expression fell. However, LGG treatment adjusted these changes and significantly enhanced the Th17/Treg balance by effectively downregulating the expression of RORγt and upregulating the expression of Foxp3 in OVX rats.

Atopic dermatitis (AD) is a chronic and recurrent inflammatory skin disease (87). Cytokines such as IFNγ and IL-12 produced by Th1 and IL-4, IL-5, and IL-13 produced by Th2 cells are responsible for the progression of AD (88, 89). Researchers formulated a multispecies probiotic mixture containing seven bacterial strains: *B. bifidium* CBT BF3, *B. breve* CBT BR3, *L. acidophilus* CBT LA1, *L. plantarum* CBT LP3, *L. rhamnosus* CBT LR5, *L. lactis* CBT SL6, and *S. thermophilus* CBT ST3, and demonstrated that the mixture of such 7 strains of probiotics induced Treg responses in a mouse model with AD (57). Alleviation of AD seems to be associated with probiotic mixture-induced generation of CD4^+^Foxp3^+^ regulatory T cells and the decreased levels of inflammatory cytokines related to Th1 and Th2 cells. Similar to this study, an independent study also found that probiotics including *Weissella cibaria* WIKIM28 and *L. plantarum* CJLP133 were proved to alleviate AD symptoms through increasing Treg cells and inhibiting proinflammatory cytokines, both in mice models and patients (58, 59).

## 5 Delicate crosstalk between probiotics and CD8^+^ T cells in antitumoral immunity

Emerging evidence suggests that probiotic supplementation can play a significant role in promoting cancer cells apoptosis, thereby inhibiting tumor progression. Specific metabolites derived from probiotic strains play crucial roles in this process. For instance, β-galactosidase secreted by *S. thermophilus* was demonstrated to inhibit colorectal tumor cell proliferation, lower colony formation, induce cell cycle arrest, and promoted apoptosis and retarded the growth of colorectal cancer xenograft (90). In addition, indole-3-lactic acid, produced by *L. gallinarum*, can protect against intestinal tumourigenesis by promoting apoptosis of colorectal cancer cells but not normal colon epithelial cells (91). Ferrichrome produced by *L. casei* inhibited the progression of pancreatic cancer cells *via* dysregulation of the cell cycle by activating p53 (92).

However, a more potent strategy for probiotics involves enhancing the cytotoxicity of CD8^+^ T cells. CD8^+^ T cells are crucial immune effectors capable of directly recognizing and killing cancer cells. However, tumors often evade this immune surveillance by creating an immunosuppressive microenvironment that inactivates or exhausts CD8^+^ T cells (93, 94). Cancer immunotherapy, particularly immune checkpoint blockade (e.g., anti-PD-1/PD-L1, anti-CTLA-4 antibodies), aims to reinvigorate these exhausted or suppressed CD8^+^ T cells by blocking inhibitory signals, thereby restoring their anti-tumor activity and leading to durable responses in various cancers, though efficacy varies and resistance can occur (95, 96).

**Figure 5:**
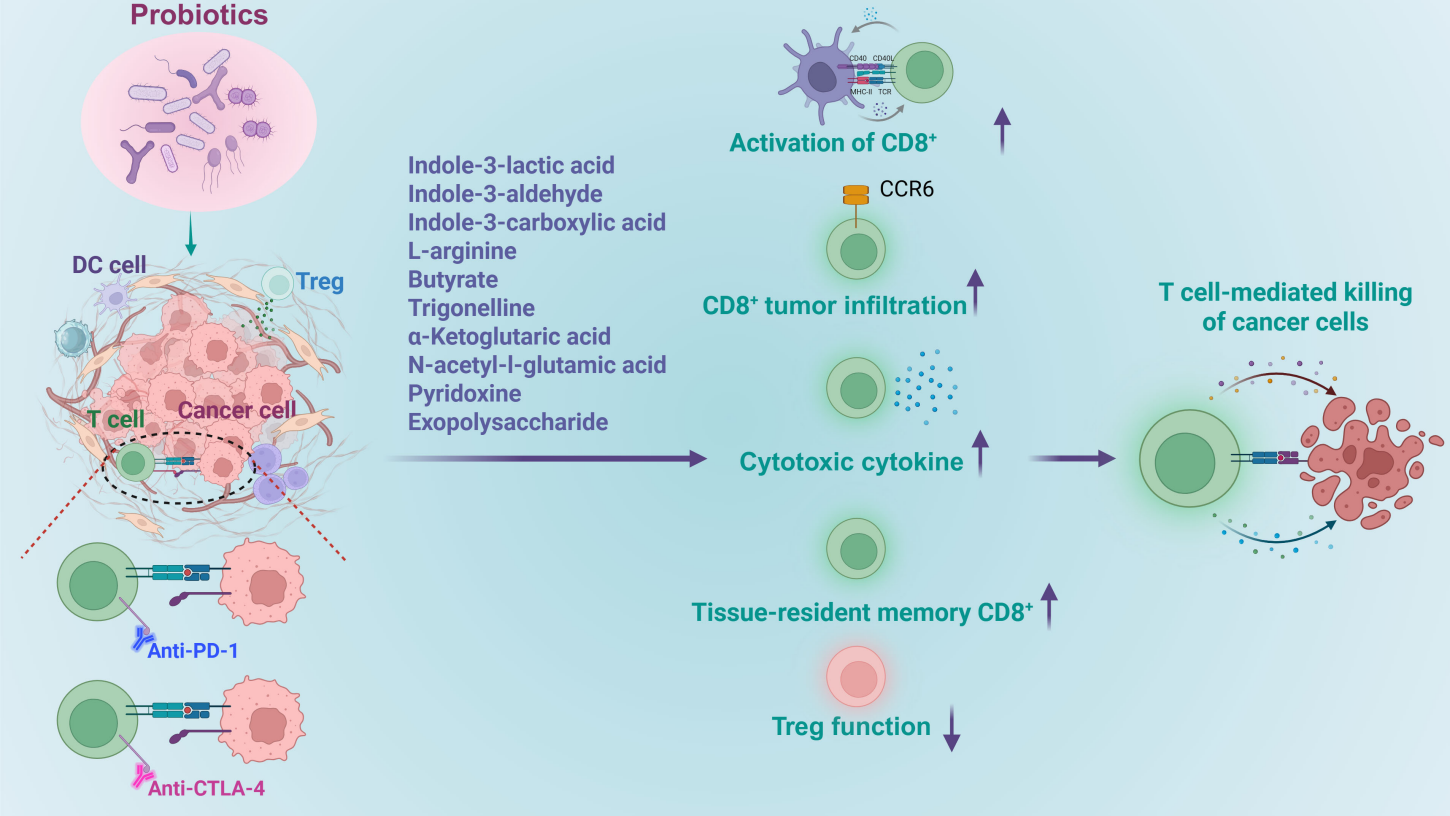
Roles of probiotics in regulation of CD8^+^ T cell mediated tumor immune therapy. Probiotics and their derived metabolites increase the efficacy of immunotherapy by boosting the activation of CD8^+^ T cells, enhancing their activation, facilitating their infiltration into tumors, promoting cytokine release, stimulating the development memory cells, while diminishing immune inhibition.

The gut microbiota is a crucial regulator of anti-tumour immunity during immune checkpoint inhibitor therapy. In the rapidly evolving landscape of cancer immunotherapy, the combination of probiotics supplementation with immune checkpoint inhibitors has emerged as a groundbreaking and innovative approach for treating a diverse array of cancers (97–99). When used alongside immune checkpoint inhibitors as an adjuvant therapeutic strategy, such probiotic bacteria were either administered through oral gavage, or intratumoral/intravenous injection to potentiate antitumor efficacy. This combination therapy holds promise for improving treatment efficacy, reducing side effects, and offering new hope for patients with various types of cancer. A number of research confirmed that probiotics, especially strains of *Lactobacillus* and *Bifidobacterium*, and their derived metabolites, can augment the efficacy of immunotherapies, holding promise in the prevention and treatment of various types of cancer types, as detailed below and shown in **Figure 5**.

### 5.1 Probiotics enhance the activation of CD8^+^ T cells

In a mouse model of colorectal cancer (CRC), *Lactobacillus* species have exhibited potential in alleviating disease progression (60). Notably, the administration of *L. plantarum* L168 and its metabolite, indole-3-lactic acid, has proven effective in mitigating intestinal inflammation, tumor growth, and gut dysbiosis. From a mechanistic standpoint, indole-3-lactic acid has been found to enhance H3K27ac binding at the enhancer regions of IL-12a, thereby boosting IL-12a production in dendritic cells. This process is instrumental in priming CD8^+^ T cell immunity against tumor growth. In addition, indole-3-lactic acid has been shown to transcriptionally inhibit Saa3 expression, which is associated with the cholesterol metabolism of CD8^+^ T cells. It achieves this by altering chromatin accessibility, which subsequently serves to enhance the function of tumor-infiltrating CD8^+^ T cells. Collectively, these findings shed new light on the epigenetic regulation of probiotics-mediated anti-tumor immunity and suggest that *L. plantarum* L168 and indole-3-lactic acid may hold potential for the development of therapeutic strategies for CRC patients.

### 5.2 Probiotics facilitate the infiltration of CD8^+^ T cells to tumor microenvironment

Researchers found that administration of *L. delbrueckii subsp. bulgaricus* OLL1073R-1 (EPS-R1) augmented antitumor effects of anti-CTLA-4 or anti-PD-1 monoclonal antibody against CCL20-expressing tumors in mice (20). This improvement was achieved by increased infiltration of CCR6^+^ CD8^+^ T cells which produced more IFNγ. Importantly, the antitumor boost conferred by EPS-R1 was independently of host gut microbiota. Mechanistic studies revealed that the phosphorylated structure in EPS-R, not the live bacteria or their metabolites, engages a lysophosphatidic acid receptor on CD8^+^ T cells, which further induced the expression of CCR6 in CD8^+^ T cells.

Supplementation of *Blautia coccoides* (*B. coccoides*) was also found to enhance the infiltration of CD8^+^ T cells into the tumor microenvironment, but with a distinct mechanism. It was known that clinical responders to cancer immunotherapy consistently harbor a gut microbiota enriched in *Blautia* (61). In a murine model of bladder cancer, supplementation with *B. coccoides* replicated this advantage: tumor growth was markedly restrained and accompanied by enhanced infiltration of CD8^+^ T cells into the tumor microenvironment (100). Untargeted metabolomics and mechanistic studies demonstrated that administration of *B. coccoides* elevated the level of trigonelline, which further downregulated β-catenin expression and promoted the infiltration and antitumor activity of CD8^+^ T cells.

### 5.3 Probiotics promote the releasing of cytotoxic cytokines by CD8^+^ T cells

Another study reveals a critical microbial-host crosstalk between *Lactobacillus*-released aryl hydrocarbon receptor (AhR) agonist indole-3-aldehyde (I3A) and CD8^+^ T cells within the tumor microenvironment (17). *L. reuteri* was identified to migrate to, establish colonization in, and persist within melanoma tumors. Through the release of the dietary tryptophan metabolite I3A, it locally stimulates the production of IFNγ by CD8^+^ T cells, thereby enhancing the efficacy of immune checkpoint inhibitors (ICIs). Moreover, *L. reuteri*-secreted I3A was both necessary and sufficient to drive antitumor immunity, and loss of AhR signaling within CD8^+^ T cells abrogated *L. reuteri’*s antitumor effects. Further, a tryptophan-enriched diet potentiated both *L. reuteri-* and ICI-induced antitumor immunity, dependent on CD8^+^ T cell AhR signaling. In conclusion, this study provided evidence for a potential role of *L. reuteri* and its metabolite I3A in promoting ICI efficacy and survival in advanced melanoma patients.

Besides *Lactobacillus* and *Bifidobacterium*, some other species are also able to boost immunotherapy through regulating the cytotoxicity of CD8^+^ T cells. It was found that *Roseburia intestinalis* (*R. intestinalis*) was substantially reduced in stool samples of patients with CRC compared to healthy individuals (19). Study on mice model revealed that administering *R. intestinalis* significantly curbed CRC tumor formation, whereby butyrate produced by *R. intestinalis* emerged as the key functional metabolite (19). Both *R. intestinalis* and butyrate were shown to suppress tumor growth in orthotopic MC38 and CT26 mouse models by activating cytotoxic CD8^+^ T cells, enhancing the production of granzyme B, IFNγ and TNFα. Specifically, butyrate directly binds to TLR5 on CD8^+^ T cells, activating NF-κB signaling to enhance T cell activity, and boosts the efficacy of anti-PD-1 therapy in mice models.

### 5.4 Probiotics stimulate the development of tissue-resident memory CD8^+^ T cells

*Bifidobacterium* supplementation is emerging as another microbiome-based adjunct therapeutic strategy for CRC. Fasting-mimicking diet (FMD) was first shown to boost antitumor immunity in CRC patients (101, 102). Metagenomic profiling of FMD-treated CRC mice revealed a selective enrichment of *B. pseudolongum*. Replenishing this species recapitulated the benefit: L-arginine, a signature metabolite of *B. pseudolongum*, rose sharply and drove the differentiation of tissue-resident memory CD8^+^ T cells (TRM) in both CRC mice and patients. Consequently, FMD or *B. pseudolongum* alone potentiated anti-CTLA-4 therapy in orthotopic CRC models. More importantly, high abundance of CD8^+^ TRM and *B. pseudolongum* was associated with a better outcome in CRC patients. In conclusion, *B. pseudolongum* contributes to the FMD antitumor effects in CRC by supplying L-arginine, which promotes CD8^+^ T-cell differentiation into memory cells (62).

### 5.5 Probiotics inhibit immune suppression function to accelerate inflammation

Other than regulating CD8^+^ T cell activity, *Lactobacillus* species can modulate the immune suppressive Treg cells, thereby affect antitumor efficacy indirectly. One of the examples is *L. gallinarum*, which can significantly improve anti-PD1 efficacy in mouse MC38 and CT26 CRC tumorigenesis models (63). Specifically, *L. gallinarum* synergized with anti-PD1 therapy by reducing Foxp3^+^ Treg intratumoral infiltration, and enhancing effector function of CD8^+^ T cells. *L. gallinarum*-derived indole-3-carboxylic acid (ICA) was identified as the functional metabolite. Mechanistically, ICA inhibited indoleamine 2,3-dioxygenase (IDO1) expression, therefore suppressing kynurenine (Kyn) production in tumors. ICA also competed with Kyn for binding site on aryl hydrocarbon receptor (AHR) and antagonized Kyn binding on CD4^+^ T cells, thereby inhibiting Treg differentiation *in vitro* and *in vivo*.

Moreover, *L. acidophilus* cell lysates was found to enhance the antitumor activity of CTLA-4 blockade when combined with an anti-CTLA-4 antibody in syngeneic BALB/c mouse models of CRC (64). Notably, unlike CTLA-4 monotherapy, the co-administration of *L. acidophilus* lysates provided significant protection against CRC development, characterized by increased CD8^+^ T cells and effector memory T cells (CD44^+^CD8^+^CD62L^+^) and decreased immunosuppressive Tregs and M2 macrophages (F4/80^+^CD206^+^).

*L. rhamnosus* Probio-M9 (Probio-M9) offers a further case in this point. Whether given prophylactically or therapeutically, Probio-M9 intervention was found to boost the anti-PD-1-based tumor inhibition, not only by enhancing cytotoxicity of CD8^+^ T cells but also by suppressing the function of Treg cells (65). In addition to promoting beneficial microbes (e.g., *Lactobacillus* and *Bifidobacterium animalis*), Probio-M9 orchestrates a metabolite milieu that favors antitumor immunity. Specifically, there was accumulated butyric acids in the gut, as well as blood-derived α-ketoglutaric acid, N-acetyl-l-glutamic acid and pyridoxine. These molecules together dampened the function of Treg cells while enhancing the infiltration and activation of CD8^+^ T cell.

A recent study showed that gut microbiome enhances antitumor immunity by decreasing PD-L2 expression on dendritic cell and its interaction with repulsive guidance molecule b (RGMb). This mechanism is particularly linked to *Coprobacillus cateniformis* (*C. cateniformis*), as colonization of *C. cateniformis* alone can significantly downregulate PD-L2 expression on dendritic cells *in vivo* and *in vitro*, thereby improving the efficacy of PD-1 inhibitors (66).

In summary, supplementation of probiotics and their derived metabolites appear to be a promising strategy for enhancing the efficacy of immunotherapy. This is achieved by boosting the activation of CD8^+^ T cells, enhancing their activation, facilitating their infiltration into tumors, promoting cytokine release, stimulating the development memory cells, while diminishing immune inhibition. Nevertheless, there are still significant challenges impede their translation into viable cancer therapies. First, probiotic benefits are highly strain-specific, demanding precise identification of effective strains for distinct cancer types. Second, safety concerns regarding probiotic supplementation in immunodeficient or immunocompromised cancer patients warrant rigorous evaluation. Third, the long-term therapeutic efficacy is yet to be conclusively established, and scaling production for widespread clinical application poses considerable difficulties.

## 6 Engineered probiotic-based therapeutic approaches for T cell mediated solid tumor targeting

**Figure 6:**
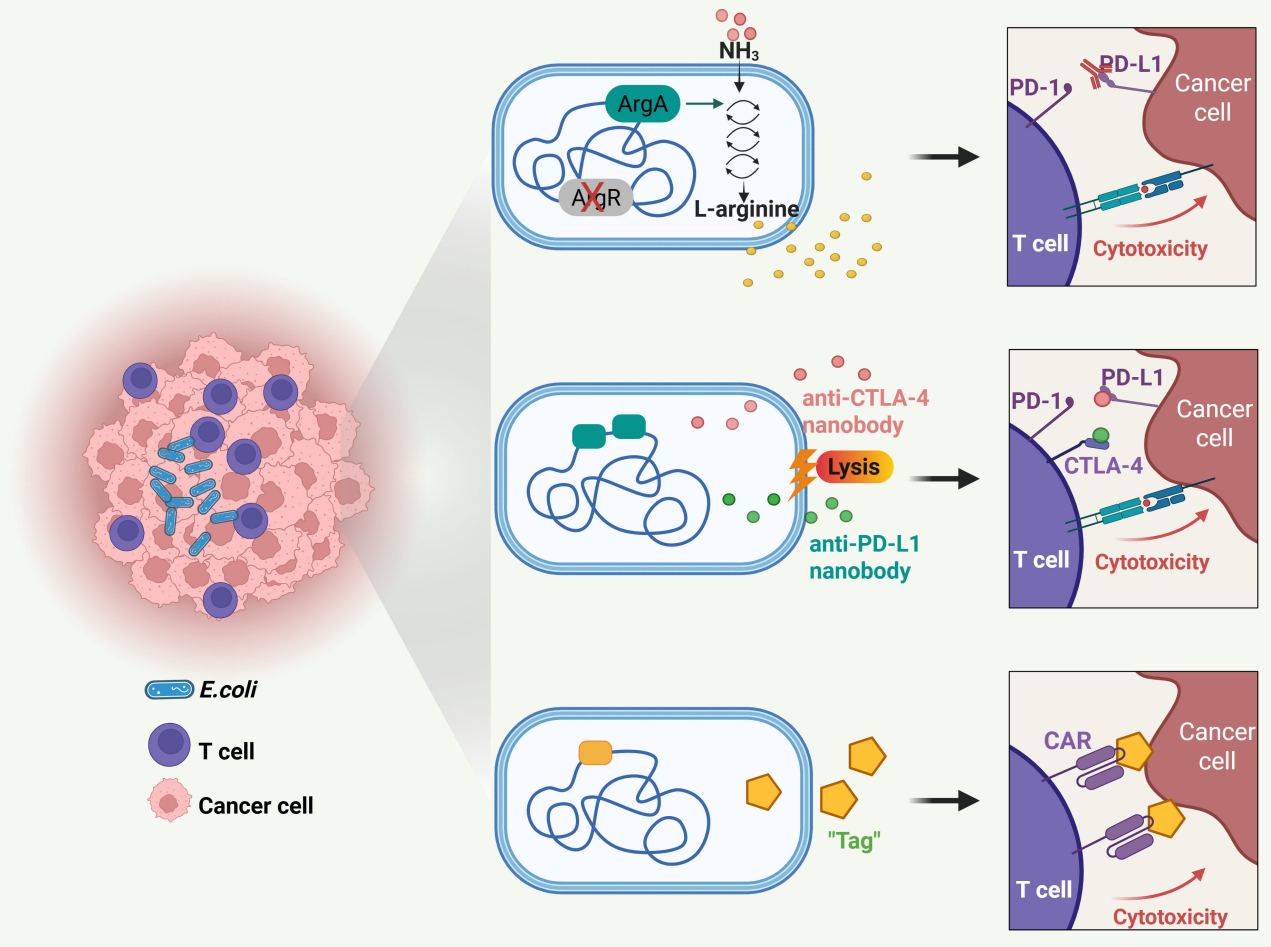
Probiotic-based novel therapeutic approaches for T cell mediated solid tumor targeting. Engineered probiotics modulate metabolic microenvironment, delivery checkpoint blockade nanobodies, and guide CAR-T cells for solid tumor targeting.

Certain bacteria, particularly those capable of surviving and thriving within the unique microenvironment of tumors, have emerged as promising tools for enhancing CD8^+^ T cell-based immunotherapy (103–105). Among such bacteria, *Escherichia coli* (*E. coli*), with a well-established human safety record, is emerging as a favored chassis for engineering “smart microbes” that deliver therapeutic modalities to the sites of cancer. By strategically engineering *E. coli*, researchers aim to harness its natural abilities to boost the efficacy of T cell-mediated anti-tumor responses, potentially leading to more effective and targeted cancer treatments, as shown in **Figure 6**.

### 6.1 Metabolic modulation of tumors with engineered probiotics for immunotherapy

It is known that the availability of L-arginine within tumors plays a pivotal role in shaping the efficacy of anti-tumor T cell responses (106, 107). Consequently, increases of typically low L-arginine concentrations within the tumor may greatly potentiate the antitumor responses of immune checkpoint inhibitors, such as PD-L1-blocking antibodies. However, presently there are no established methods to raise intratumoral L-arginine locally. In light of the fact that bacteria can survive and thrive in tumors, a recent study leveraged a non-pathogenic strain of *E. coli*, *E. coli* Nissle 1917 (ECN), as an intratumoral, synthetic biology-based cellular therapy to generate high local concentrations of arginine (67). Through deleting the arginine repressor gene (ArgR) and integrating the N-acetylglutamate synthase gene (ArgA) in ECN, researchers engineered the bacteria to efficiently channel ammonia toward arginine synthesis. The findings revealed that when these engineered bacteria colonized tumors, they increased intratumoral L-arginine levels, expanded the population of tumor-infiltrating T cells, and synergized remarkably with PD-L1 blocking antibodies in the clearance of tumors. These results indicate that engineered microbial therapies can modulate the tumor microenvironment metabolically, thereby augmenting the efficacy of immunotherapies.

### 6.2 Engineered probiotics for local tumor delivery of checkpoint blockade nanobodies

As discussed in Section 6, while immune checkpoint inhibitors targeting PD-L1 and CTLA-4 have transformed cancer immunotherapy, they can cause immune-related adverse effects like fatigue, skin rashes, endocrine disorders, and hepatic toxicities (108, 109). Therefore, there is a need for improved delivery methods to provide localized, sustained, and minimally invasive treatment. In this situation, *E. coli* was demonstrated a preferred tool for local delivery of PD-L1 and CTLA-4 antibodies (68). Researchers designed an engineered *E. coli*-based system to deliver checkpoint blockade nanobodies to tumors. The system, equipped with an optimized lysing mechanism, allows probiotic bacteria to colonize the tumor core, grow, and release nanobodies continuously. A single injection of this system led to tumor regression in mouse models, increased activated T cells and enhanced T cell memory populations, supporting a potentiated systemic immune response.

### 6.3 Probiotic-guided CAR-T cells for solid tumor targeting

Furthermore, probiotics can direct chimeric antigen receptor (CAR)-T cells to exert enhanced cytotoxicity within the solid tumor microenvironment (69). CAR-T therapy, based on T cells genetically engineered to express tumor-targeting receptors, has revolutionized the treatment of hematologic cancers (110, 111). However, its efficacy against solid tumors remains limited (112, 113). This is due to the absence of unique surface antigens on solid tumor cells, which poses a major obstacle to identifying optimal targets for therapy and developing new CARs (114–116). Conversely, specific bacteria selectively colonize tumor cores and can be engineered into antigen-independent platforms for therapeutic delivery. A recent study demonstrated this through a novel two-step strategy (69). First, an engineered nonpathogenic *E. coli* strain delivers synthetic antigens to the tumor microenvironment, effectively “tagging” the tumor cells. Next, CAR-T cells are specifically designed to recognize these synthetic antigen tags. Upon administration of the *E. coli* probiotic, the engineered CAR-T cells are directed to the tagged solid tumors. This system successfully orchestrated tumor cell killing and was demonstrated safe and effective in multiple xenograft and syngeneic models of human and mouse cancers.

While challenges remain, engineered bacteria represent a promising frontier in oncotherapy. These bacteria leverage their natural tropism to selectively target tumors, delivering cytotoxic payloads directly to cancer cells while minimizing damage to healthy tissues. Additionally, they can modulate the immune system, enhancing the body’s natural defenses against cancer and overcoming treatment resistance that often arises in conventional therapies. As research in synthetic biology, microbiome engineering, and biomaterial integration advances, bacterial therapies may become integral to personalized cancer regimens, offering tailored treatment options that maximize efficacy and minimize side effects.

## 7 Conclusion

The interaction between probiotics and T cell-mediated immunity is highly intricate, involving a complex network of signaling pathways and molecular mechanisms. These interactions collectively shape the overall immune response, influencing whether the immune system responds appropriately to pathogens while maintaining immune homeostasis to prevent excessive inflammation. The balance achieved through this crosstalk is critical, as it determines the body’s ability to combat infections and tumors while avoiding autoimmune or inflammatory disorders.

Although preclinical studies and clinical observations have indicated that probiotics hold potential promise in various applications, several case reports, clinical trials, and experimental models have highlighted theoretical risks associated with probiotic use. These risks encompass systemic infections, which may occur if probiotics translocate across the gut barrier into the bloodstream or other sterile sites, particularly in immunocompromised individuals. Adverse metabolic activities could arise if probiotics produce harmful metabolites or disrupt the host’s metabolic homeostasis. Additionally, probiotics might overstimulate the immune system in susceptible individuals, leading to exaggerated inflammatory responses. The potential for gene transfer between probiotics and other microorganisms, especially concerning antibiotic resistance genes, is another concern.

Thus, to fully harness the benefits of probiotics while mitigating these risks, further research is imperative. An in-depth insight is needed into the specific interactions between probiotics and T cells, which are pivotal in immune regulation. Identifying the most effective probiotic strains and optimizing formulations for different clinical scenarios will be crucial steps toward maximizing their therapeutic potential.
